# Assessing the efficacy of vaginal hyoscine butyl bromide on cervical ripening prior to intrauterine procedures: A double-blinded clinical trial

**Published:** 2016-11

**Authors:** Shiva Hadadian, Masoumeh Fallahian

**Affiliations:** *Preventative Gynecology Research Center, Shahid Beheshti University of Medical Science, Tehran, Iran.*

**Keywords:** *Butylscopolammonium bromide*, *Cervical dilatation*, *Cervical ripening*

## Abstract

**Background:**

Unripe cervix prevents entering the endometrial cavity during intrauterine procedures. Mechanical dilatation of cervical canal might cause undesirable complications.

**Objective:**

To investigate the substitute of mechanical intervention with chemical treatment by administering hyoscine to patients.

**Materials and Methods:**

Sixty non-pregnant women, 20-70 years of age, with a closed cervix who were scheduled for an intrauterine procedure, were randomly divided into two groups. Group A as experimental (received two doses of hyoscine) and group B, as control group (received two doses of vitamin B6) in the vagina (8 hrs and 2 hrs before procedure) and the effect of these two drugs on dilatation and consistency of cervix were studied.

**Results:**

Statistics resulted from Mann-Whitney U test (p=0.027) and ^2^(p=0.002) indicated that in premenopausal women, the priming effect of hyoscine on dilatation and consistency of uterine cervix was significant, but there were no significant benefits from giving vaginal hyoscine to menopausal women preoperatively (p=0.603).

**Conclusion:**

Hyoscine proved a good choice for inducing cervical priming before intrauterine procedures in premenopausal women.

## Introduction

Unripe cervix has been complained as the main and frequently encountered barrier that impedes entering the endometrial cavity during intrauterine procedures in cases such as diagnostic dilatation and curettage, endometrial biopsy with Pipelle, hysteroscopy, endometrial ablation, intrauterine insemination (IUI), intrauterine device (IUD)s insertion, sono hysteroscopy, etc. Traditional dilatation of cervix with Hegar dilators may not be possible in some patients with very tight or abnormal cervix. Besides, mechanical dilatation of cervical canal might cause uterine perforation, cervical tearing, bleeding, producing a false passage, incompetency of cervix or scarring, and subsequent cervical stenosis.

Cervical priming with a chemical agent, before intrauterine procedures, makes the operation easier and decreases the incidence of complications. Saving the operation time and using a lower dose of anesthesia drugs are other advantages (1). Research has shown that cervical injury and uterine perforation can be prevented via preoperative cervical ripening during termination of pregnancy (2).

In 1985 trials of cervical priming began and before diagnostic hysteroscopy intracervical Sulprostone Gel was applied. This cervical priming led to a reduction in the force required for cervical dilatation (3). In 2005, Bancaite *et al. *found that using vaginal misoprostol, before hysteroscopy, reduced cervical resistance (4). The results of these studies were compatible with the study conducted in 2014 which showed that vaginal misoprostol applied to non-pregnant women, before dilatation and curettage, facilitated the cervical dilatation and minimized cervical or uterine injuries (1). 

But, the side effects of prostaglandins (nausea, vomiting, diarrhea, and fever) are obstructive elements of their use, and the use of misoprostol has some contraindications such as intrauterine device (5). Besides, as misoprostol is expensive, it is usually saved for special cases. Therefore, the researchers decided to investigate the use of hyoscine, in case, there is no access to misoprostol or the use of it is contraindicated. Since hyoscine has spasmolytic effect, it facilitates the dilatation of cervix with lower complications and is easily available in the market.

## Materials and methods

The present study was carried out in the Department of Obstetrics and Gynecology at Sirjan Gharazi Hospital in Kerman, Iran, from 2014 to 2015. It was approved by the Ethics Committee of Shahid Beheshti University of Medical Science (IR.sbmu.sm.rec.1394.82). Before enrollment, the participants were told all about the purpose and course of the study and they signed the informed consent forms. The study was designed to be double blinded.

In this clinical trial, which was controlled by placebo, sixty non-pregnant women (20-70 years of age) with general good health and stenotic cervix were randomly divided into two parallel groups. These patients were scheduled for intrauterine procedures because of gynecological diseases or IUD insertion. Patient selection for this study was based on history and examination of the participants. The patients with a history of injury to the cervix, incompetence of the cervix, cervical surgery, hypersensitivity to hyoscine and those suffering from glaucoma, cardiovascular disease, and tachycardia (heart beat rate >100) were not included in this study. Also, the patients who were on hypertension drugs or estrogen replacement therapy were eliminated from this study. 

Group A, as the experimental group, received two doses of hyoscine (tab 10 mg Ramofarmin Company, Iran). Group B, as a control group, received two doses of vitamin B6 (tab 40 mg Ramofarmin Company, Iran). The tablets were placed in the posterior fornix of the vagina (8 hr and then 2 hr before the procedure).

Vitamin B6 was selected as a placebo due to the fact that it doesn’t have any significant systemic effects when we use it vaginally and, it has no effect on cervical tissue or uterine (6, 7). Also, the similarity between hyoscine and vitamin B6 in shape, size, and color makes them hardly distinguishable from each other. The tabs were placed in identical envelopes with different code numbers. Neither the investigator nor the patients knew which tab was taken. In this way, the effects of these two drugs on dilatation and consistency of cervix were studied. If the vaginal examination, before the intrauterine procedure, showed that the drug had not been dissolved, and it was seen in the vagina, the patient was excluded from the study.

In qualified patients, cervical responses to the drugs were determined in two ways, one by subjective assessment of the consistency of the cervical tissue and the other by measuring the patency of the cervix. This measurement was performed by using reversed dilator method, starting from number 8 (8mm) Hegar dilator. If the first dilator didn’t pass through the cervical os, smaller ones were successively tried, and the largest Hegar dilator diameter that passed through the cervix without resistance was considered as secondary cervical dilatation. Side effects such as nausea, vomiting, tachycardia or dry mouth were recorded. 

**Statistical analysis**

The data was analyzed using SPSS 22; and Chi-square test, Mann-Whitney U test, and T-test were used to compare the results. Significance levels for tests were determined as 95% (p<0.05).

## Results

**Figure 1:**
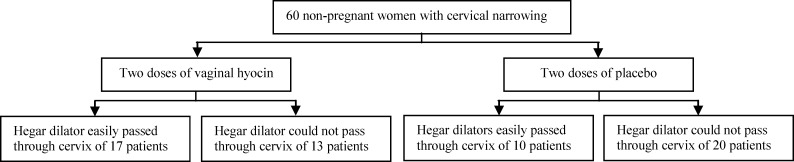
A flow chart demonstrates effect of Hyoscine on cervical ripening is more than the placebo

The average age of this study participants was 41.3±13.7 years in hyoscine group and 42±13.5 years in vitamin B6 group. Mann-Whitney U test indicated that there was not any significant difference between the average age of the two groups (p=0.8). Nine patients (30%) in hyoscine group and eleven patients (36.7%) in vitamin B6 group were menopause. However, ^2^ test pointed out that the two groups were homogenous regarding menopausal status (p=0.584). These two groups were comparable regarding their parity and their type of birth delivery (p=0.68) (Mann-Whitney U test). Hegar dilator number six ultimately passed through the cervical canal in 17 (56.7%) patients of hyoscine group and ten patients (33.3%) of placebo groups (Figure 1). 

Cervical os in the hyoscine group (3.93±0.65) was wider than the control group (2±0.53). The difference between the two groups was statistically significant (p=0.017). So the use of vaginal hyoscine facilitates dilatation of the cervix. Chi-square test pointed out that the consistency of cervical tissue after intervention in hyoscine group was statistically different (p=0.001) from control group which means hyoscine ripens the cervical tissue. Following the application of the drugs in the two groups, the cervical width for premenopausal women was 4.76±3.55 mm in hyoscine group and 2.53±3.04 mm in control group (p=0.603). Mann-Whitney U test pointed out that the difference in cervical width after intervention in postmenopausal women was not statistically meaningful. So the drug was effective only for premenopausal women. Hyoscine had minor side effects such as tachycardia, dry mouth, and flushing (3, 5, 6). However, the difference in side effects between hyoscine group and placebo group was not significant.

**Table I:** Demographic finding between two groups

**Parameters**		**Hyoscine**	**Vitamin B6**	**p-value**
Age (years, mean± SD)		41.3 ± 13.7	42 ± 13.5	0.8
Menopausal status [n (%)]				
	Premenopausal women	21 (70)	19 (63.3)	0.584
	Menopausal women	9 (30)	11 (36.7)	0.584
	Vaginal delivery	14 (46.7)	16 (54.4)	0.68

Informations gathered via questionnaire

**Table II:** Side effects of the drugs

**Side effects [n (%)]**	**Hyoscine**	**Vitamin B6**	**p-value**
Tachycardia	5 (16.7)	3 (10)	0.706
Dry mouth	6 (20)	2 (6.7)	0.254
Flushing	3 (10)	3 (10)	1

Informations gathered via questionnaire

**Figure 2:**
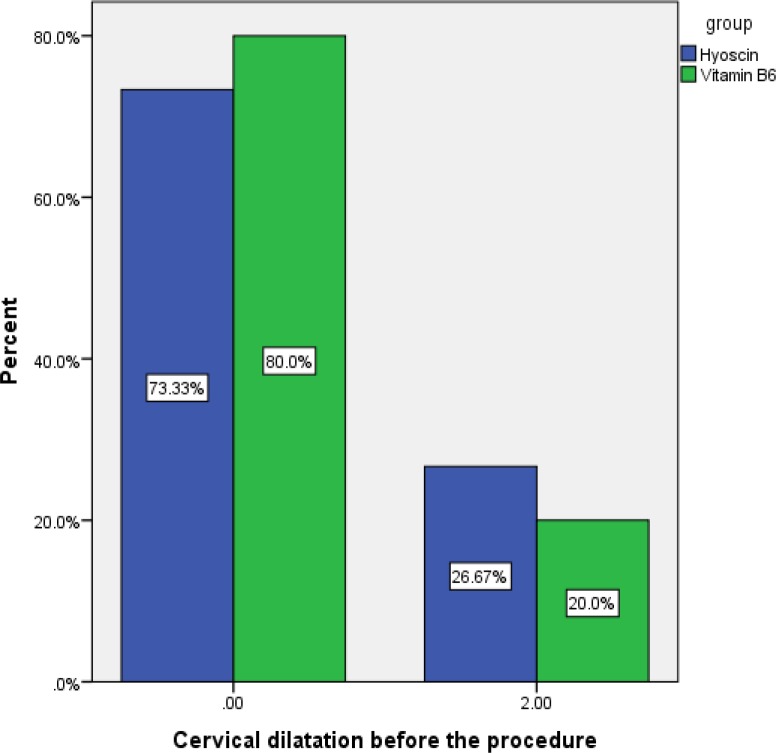
Cervical dilatation before the procedure

**Figure 3:**
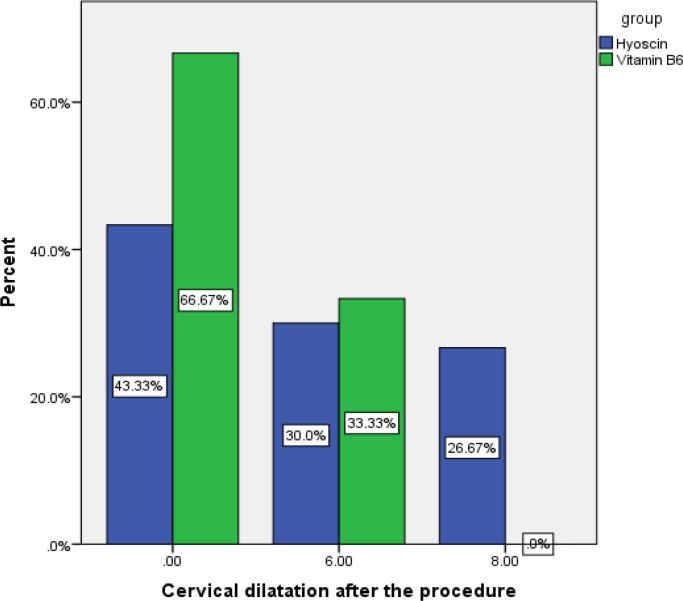
Mann-Whitney U Test analyzes Cervical dilatation after the procedure

## Discussion

Cervical dilatation represents a challenge for the time when intrauterine procedures in women with cervical narrowing is due to happen. Although the mechanism of cervical ripening has not been clearly defined, it has been suggested that pro-inflammatory cytokines, prostaglandins, relaxin, and gonadal steroid regulate connective tissue remodeling which might play a role in this process. Inhibitory impulses, in the form of spasm, often impair the dilatation of cervix (8-10). [Spasmolytic drugs are frequently employed to overcome cervical spasm. Thus, they reduce the duration of labor. One of these spasmolytic drugs is hyoscine butylbromide, which has been used to shorten the duration of labor] (11, 16). Several studies have shown that intravenous application of hyoscine butylbromide during the active phase of labor increases cervical dilatation (12, 13). Because of the beneficial effects of hyoscine on cervical ripening of pregnant women, it has been hypothesized that hyoscine is a good choice for non-pregnant women because the dilatation of cervix might be facilitated. 

‘‘Hyoscine butylbromide, under the trade name of Buscopan, belongs to a parasympatholytic group of drugs, and it is a semisynthetic derivative of Scopolamine’’ (14). It has effective anti-spasmolytic activity with a half-life of 4.5-5 hrs. According to Obama *et al, *hyoscine butylbromide has a selective action on cervicoutrine plexus and brings about cervical dilatation (14, 15). Based on New Zealand Database, adverse effects of hyoscine after oral usage include skin reaction, tachycardia, vascular disorders (Buscopan injection), dry mouth, urinary retention, and gastric irritation which are dose dependent. These effects could be decreased by administering the tablet vaginally. Because of the rich blood supply of vagina, the use of the drug vaginally will substantially increase its effect on the cervix. Although the use of the drug in the vaginal route has a lot of benefits including the easy application of the drug by the patients, the effect of hyoscine given in this way has rarely been studied. 

This study pointed out that vaginal hyoscine was effective only in premenopausal women and, unfortunately, post-menopausal patients didn’t get any clear benefit from using it pre-operatively. Similar to the results of misoprostol studies, the result obtained here, may be due to either the change in cervical tissue collagen component or the deficiency of gonadal steroids (16). Other studies are needed to investigate the effect of hyoscine on post-menopausal women pretreated with vaginal estrogen.

## Conclusion

Vaginal hyoscine proved a good choice for inducing cervical priming in non-pregnant premenopausal women before the intrauterine procedure, but its efficacy is not significant in postmenopausal women.

## Note

***Registration ID in*** ***IRCT*** ***: IRCT2015121312190N5***
